# Enzymatic analysis of the effect of naturally occurring Leu138Pro mutation identified in SHV β-lactamase on hydrolysis of penicillin and ampicillin

**DOI:** 10.1186/1471-2180-11-29

**Published:** 2011-02-04

**Authors:** Nabin Rayamajhi, Jeong Chan Joo, Seung Bin Cha, Subarna Pokherl, Min Kyung Shin, Young Je Yoo, Han Sang Yoo

**Affiliations:** 1 Department of Infectious Diseases, College of Veterinary Medicine, KRF Zoonotic Disease Priority Research Institute, Brain Korea 21 for Veterinary Science, Seoul National University, Seoul 151-742, S. Korea; 2 School of Chemical and Biological Engineering, Seoul National University, Seoul 151-744, S. Korea

## Abstract

**Background:**

The aim of this study was to analyze the significance of leucine to proline substitution at position 138(Leu138Pro) on the hydrolysis of penicillin and ampicillin that we identified in the *bla*_SHV _gene of clinical *Escherichia coli *swine isolate.

**Results:**

Kinetic analysis of the mutant proteins showed that *K*_*m *_value of the purified L138P mutant was comparatively higher than SHV-1, SHV-33 and SHV-33(L138P) enzyme for penicillin and ampicillin. Docking simulation of the SHV-1 and SHV-(L138P) enzymes also confirmed that β-lactamases preferred penicillin to ampicillin and the SHV-1 had a higher binding affinity for antibiotics compared to the SHV-(L138P) and other mutants.

**Conclusions:**

Our result demonstrated that L138P has a reduced role in penicillin and ampicillin hydrolyzing properties of SHV β-lactamases. These naturally occurring mutations rendering reduced function of the existing protein could trigger the emergence or acquisition of more effective alternative mechanisms for β-lactam hydrolysis.

## Background

Antimicrobial resistance based on hydrolysis of the antibiotic by β-lactamases is currently a worldwide problem. It is one of the single most prevalent mechanisms responsible for resistance to β-lactams in clinical isolates of the *Enterobacteriaceae *[1-3]. Among the four classes (A to D) of β-lactamases, plasmid mediated class A and C β-lactamases have been of high clinical concern in hospital as well as community acquired infections [1,4]. Promiscuous plasmids carrying β-lactamase encoding genes are described to spread drug resistance among different groups of microbes under local selection pressure imposed by the commonly used antibiotics [1,5,3]. One of the most common plasmid mediated β-lactamase enzymes is closely related to TEM and SHV penicillinase [6,3]. Recently CTX-M and AmpC type β-lactamase are being widely reported from Enterobacteriaceae that are associated with nosocomial and community acquired infections [1,7].

Use of extended-spectrum β-lactam antibiotics has led to the occurrence of variants of these β-lactamases carrying amino acid substitutions that alter the enzyme's substrate specificity [1,6,8,9]. SHV-1 is an important plasmid mediated β-lactamase found in the chromosome of most strains of *Klebsiella pneumonia. *Its hydrolytic spectrum of activity is similar to that of TEM -1, but it shows better activity against ampicillin [10,11]. Natural evolution and appearance of mutations has taken place in response to an array of different penicillin derivatives, cephamycins and fourth generation cephalosporins. After identification of SHV-2, the first plasmid-mediated β-lactamase capable of hydrolyzing extended-spectrum cephalosporins, several point mutations in SHV β-lactamase have been reported that altered the architecture of the active site of the enzyme [8,12-14]. This modification leads to either an increase in minimum inhibitory concentration (MIC) or broadens the spectrum of the antimicrobial resistance observed. Amino acids from the region around the position 182 to the catalytic triad do not generally tolerate substitution in TEM β-lactamase and are thought to be necessary for proper core packing and catalytic residue orientation [15,9]. Highly conserved residues on Class A β-lactamases (Phe 66 and Pro 67) are involved in hydrophobic core packing interactions. Likewise Thr 71 and Lys 73 are important for proper positioning of the catalytic residues Ser 70 and Asn 132 [16,13]. However, the effect of substitutions on amino-acid residues that alter the substrate hydrolyzing property of SHV enzyme is still unknown. The SHV β-lactamases identified in our study contained a single L138P change compared to wild-type enzyme SHV-1. Since this mutation occurred naturally in SHV-1 β-lactamases, we speculated that any changes in the substrate affinity must be attributed to this single amino acid substitution. Thus, to gain deeper insight we performed cloning, expression and enzyme kinetics of SHV L138P β-lactamase. For uniformity and comparative study we cloned a wild type *bla*_SHV-1 _gene from *K. pneumoniae *into the pET 200 cloning and expression vector. This plasmid was used as template for creating SHV-33 and target mutant SHV alleles (*bla*_SHV-L138P, _*bla*_SHV-33(L138P)_) by site directed mutagenesis. Since SHV-33 has a single amino-acid substitution in SHV-1 and was previously identified in our study, we used these known β-lactamases as control. The phenotypic and enzyme kinetics results were also verified by a molecular docking simulation experiment.

## Methods

### Bacterial strains

*E. coli *was isolated from the feces of pigs with mixed clinical signs of digestive and a respiratory disorder was identified by biochemical tests and by VITEK (Vitek system; bioMerieux, Marcy l'Etoile, France). Once identified, the culture was stored in Tryptic Soy Broth (TSB) (Difco Laboratories, Detroit, MI) mixed with 20% glycerol (Shinyo Pure Chemicals Co. Ltd., Japan) at -70°C until use.

### Bacterial strains and antimicrobial tests

An *E. coli *isolated from the feces of pigs with mixed clinical signs of digestive and a respiratory disorder was tested with antibiotic susceptibility discs according to the guidelines of the Clinical and Laboratory Standards Institute (CLSI) [17]. The active ingredients of the selected antibiotics were cefotaxime (CTX), ceftazidime (CAZ), cefoxitin (FOX) and ceftiofur (CEF). The isolate was further tested by the double disk diffusion tests using cefotaxime (CTX), ceftazidime (CAZ), cefoxitin (FOX) in combination with amoxicillin/clavulanic acid (AMC) (Becton Dickinson, Germany) and Oxoid Ltd., UK) [17]. The MIC_S _were determined by micro broth dilution method for the cephalosporins that showed complete or decreased inhibition zone diameter in the disk diffusion test. Performance and evaluation of the MIC determinations followed the recommendation of the CLSI [18].

### Sequence analysis of the β-lactamases genes

Oligonucleotide primers targeting TEM and SHV β-lactamases and sequencing of the PCR products was performed as described in our previous study [5]. The search for the homologous sequence was conducted in the GenBank database using the Basic Local Alignment Search Tool (BLAST) through the National Center for Biotechnology Information (NCBI) web site (http://www.ncbi.nlm.nih.gov/BLAST). Nucleotide substitutions were analyzed based on information available in http://www.lahey.org/studies/webt.htm.

### Site directed mutagenesis of bla_SHV-1 _genes

Wild type *bla*_SHV-1 _gene from *K. pneumonia*e was cloned in pET 200 cloning vector. This plasmid was used as template for generating *bla*_SHV(L138P)_, *bla*_SHV-33(P226S) _and *bla*_SHV-33(L138P) _genes by site directed mutagenesis following the procedures described by Zheng *et. al *[8,19]. Description of the primers used in the study are listed in Table 1. All the PCR-amplified products were evaluated by agarose gel electrophoresis and the band with the expected size was extracted using QIAEX^® ^II gel extraction kit (Qiagen, Hilden, Germany) and further treated with 10 U *Dpn*I (New England, Hertfordshire, UK) and incubated at 37°C for 3 hrs. An aliquot of 2 μl of this PCR product was transformed into TOPO 10 competent cells and plated on Tryptic Soy Agar (TSA) (Difco Laboratories, Detroit, MI) agar plate containing 100 μg/ml of kanamycin. A total of 3 colonies were selected and their plasmids were extracted using mini-prep. Sequences of all these β-lactamases were confirmed twice by the nucleotide sequencing using T7 forward and reverse primers.

**Table 1:** Primers used for detection of TEM and SHV β-lactamases and for site directed mutagenesis in this study

Targets	Primer	Sequence (5'-3')	Product size(bp)	Annealing temp	Gene bank **Accession no**.
TEM	TEM-F TEM-R	TCG GGG AAA TGT GCG TGC TTA ATC AGT GAG GCA CC	1074	62	AM849806
SHV	SHV-F SHV-R	GCC GGG TTA TTC TTA TTT GTC GC ATG CCG CCG CCA GTC A	1016	62	EU342351
SHV-M^a^	SHV-MF SHV-MR	C AAT CTG CCG CTG GCC ACC GTC GGC GGC CAG CGG CAG ATT GGC GGC GCT G		52	-
SHV-33^b^	SHV-33F SHV-33R	GTG CTG TCT GCG GGC TGG TTT ATC GCC CGC AGA CAG CAC GGA GCG GAT C		52	-

^a ^: primer used to create the mutation L138P in SHV-1 β-lactamase identified in this study

^b: ^primer used to create a single mutation P226S (SHV-33 β-lactamase)

### β-lactamase expression and Western blot

The expression of four different recombinant proteins encoding in the pET 200 expression vector carrying *bla*_SHV-1_, *bla*_SHV-1(L138P), _*bla*_SHV-33 _and *bla*_SHV-33(L138P) _genes was carried out in Rossetta-gami (RG) *E. coli *cells. The cells pellets harvested by centrifugation were washed with PBS twice, re-suspended in lysis buffer (20 mM imidazole) overnight at 4°C and lysed by sonication. The His Spin Trap (GE Healthcare, Buckinghamshire, UK) were used for elution of the protein by 500 mM imidazol and protein concentrations of all β-lactamases were determined by BCA protein assay kit (Pierce, Rockford, IL) with bovine serum albumin as standard [20]. Proteins separated by SDS-PAGE (Mini-Protean II, Bio-Rad, Hercules, CA, USA) were transferred to Hybond ECL nitrocellulose membrane (Amersham life Science, Buckinghamshire, UK), and incubated in anti His mouse IgG followed by rabbit anti mouse IgG. Binding was detected using an AP conjugate substrate kit (Bio-Rad) according to the manufacture's instruction.

### Enzyme activity assay

β-lactamase activity was determined by observing the rate of penicillin and ampicillin hydrolysis at 240 nm and 235 nm, respectively. Enzyme assay was performed at 25°C in 1 mM phosphate buffer (pH 7.0) [12]. Spectrophotometric measurements were made on Analytic Jena AG (winASPECT^®^, spectroanalytical software) using 1.0-cm path length cuvette. The values for *K*_m _and *V*_max _were determined using GraFit 6 (Erithacus Software, UK).

### Molecular docking simulation

The wild-type structure of SHV (pdb code: 1shv) was used as a template for molecular modeling. All molecular modeling simulations were performed by Discovery Studio 2.5 (Accelrys, USA) and CHARMm forcefield and CFF partial charge were used for all simulations. The conformation of L138P position was optimized by the Dreiding minimization and the molecular dynamics by standard dynamics cascade protocol was applied to relax the conformations of the wild-type and L138P mutant with and default parameters except that production steps was 3000 and implicit solvent model was set to Generalized Born method. Among produced structures, the most stable structure with the lowest potential energy was selected as modeled structure for further docking simulation.

**Figure 1:**
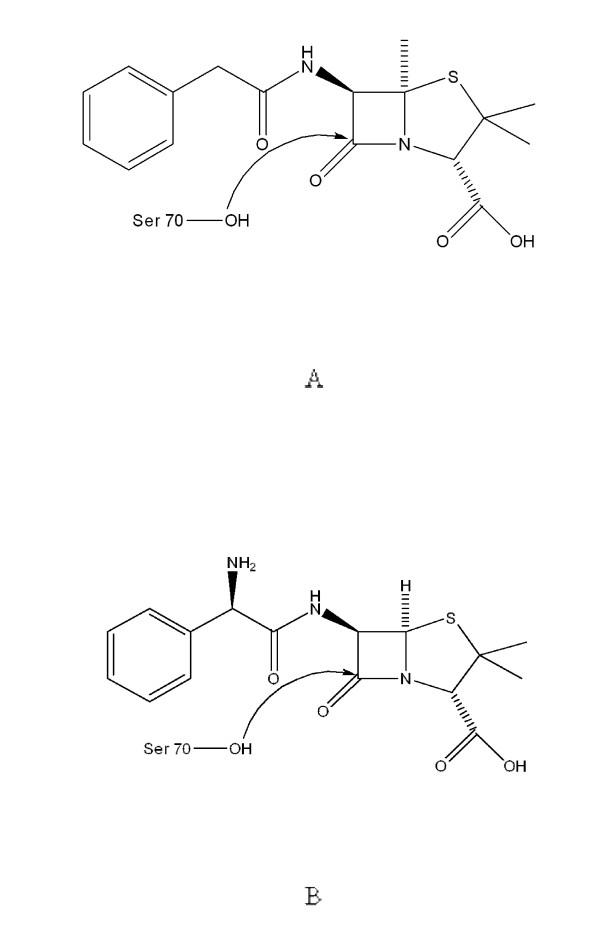
**Structures of penicillin G (A) and ampicillin (B)**.

The docking simulations of β-lactamases were conducted by CDOKER module with manually designed penicillin and ampicillin molecules. Because the active site and catalytic residues of SHV and TEM lactamasese are highly conserved, the structure of TEM with bound penicillin G (pdb code: 1fqg) was used as a reference structure to identify the initial binding site of penicillin and ampicillin in the wild-type and L138P lactamases. Once docking complexes of lactamases and antibiotics were predicted by CDOCKER module, productive docking structures of the carbonyl carbon of β-lactam ring of penicillin and ampicllin directly oriented to the OH group of catalytic S70 of β-lactamases (for nucleophilic attack of S70) were considered further binding energy calculation (Figure 1). The binding energies of the wild-type and L138P lactamases toward penicillin and ampicillin were calculated using Calculate Binding Energies protocol with default parameters except that ligand minimization were performed to consider the flexibility of residues within binding sites and implicit solvent model was set to Generalized Born method.

## Results

### Antimicrobial resistance phenotype and genotype

*E. coli *485 exhibited resistance to the commonly used antimicrobial agents on farms. The Disk diffusion test showed reduced inhibition zone diameter to cefotaxime (CTX), ceftazidime(CAZ), ceftiofur (CEF) but not to cefoxitin (FOX). This strain exhibited >5 mm increase in inhibition zone diameter of both cefotaxime and ceftazidime in the presence of amoxicillin/clavulanic acid (AMC) in contrast to when the antibiotics were tested alone. RG *E. coli *cells carrying *bla*_SHV-1_, *bla*SHV_-(L138P)_, *bla*_SHV-33 _and *bla*_SHV-33(L138P) _exhibited variable zone diameter to penicillin and ampicillin in the disk diffusion test. No decrease in zone diameter was noticed for cefotaxime (CTX), ceftazidime(CAZ), ceftiofur (CEF) and cefoxitin (FOX). The MIC values for all *E. coli *strains are listed in table 2. Genotype analysis of *E. coli *isolate showed TEM and SHV β-lactamase genes showed 100% identity to *bla*_TEM-20 _and *bla*_SHV-1 _genes except at position 138 where leucine (L) to proline (P) polymorphism was detected.

**Table 2:** Phenotype and genotype of β-lactamases for the *E. coli *field isolate and mutants included in the study

	**Inhibition Zone diameter (mm)/MICs (mg/L)**^**a**^						β-lactamases	
	
Strains	AM	PEN	CEF	FOX	CAZ	CTX	SHV	TEM
*E. coli*	≤1/640	≤1/640	8/320	15/20	11/160	12/320	SHV-1(L138P)	TEM-20
RG *E. coli-*M1	12/160	1/40	-	-	-	-	SHV-1	
RG *E. coli-*M2	28/40	14/40	-	-	-	-	L138P	
RG *E. coli-*M3	11/160	1/160	-	-	-	-	P226S	
RG *E. coli-*M4	28/20	12/2	-	-	-	-	L138P P226S	

^a^Ampicillin (AM), penicillin (PEN), ceftiofur (CEF), cefoxitin (FOX), ceftazidime (CAZ), cefotaxime (CTX)

### Site directed mutagenesis of bla_SHV-1 _genes

After cloning and confirmation of *bla*_SHV-1 _genes in the pET 200 cloning and expression vector, reverse mutation at single point (L138P) was successfully performed by site directed mutagenesis to generate *bla*_SHV-(L138P)_. Plasmid carrying *bla*_SHV-1 _gene was used to generate another mutation (S226P) that showed complete identity to *bla*_SHV-33 _gene. Sequence analysis also showed that the final site directed mutagenesis on the plasmid carrying *bla*_SHV-33 _gene, gave rise to the *bla*_SHV-33(L138P)._

### Cloning, expression and β-lactamase activity assay

All four pET 200 cloning and expression vectors carrying *bla*_SHV-1_, *bla*_SHV-1(L138P), _*bla*_SHV-33 _and *bla*_SHV-33(L138P) _genes expressed in Rossetta-gami *E. coli *cells. Expressed proteins matched the size of 32.22 kDa in SDS-PAGE and Western blot. Enzyme kinetics showed that SHV β-lactamases cloned and expressed in this study exhibited variable catalytic activity of penicillin and ampicillin. *K*_m _value for both penicillin ampicillin was lowest for SHV-1 β-lactamase followed by SHV-33, SHV-33(L138P) and SHV-L138P. The description of the *K*_*m*_, *k*_cat _and *k*_cat_/*K*_*m *_values are given are listed in table 3.

**Table 3:** Kinetics parameters for penicillin and ampicillin

	penicillin			ampicillin		
	
Enzymes	*Km*(μM)	***K*cat (s**^**-1**^**)**	*K*cat*/Km * **(**μM^**-1**^**s**^**-1**^**)**	*Km*(μM)	***K*cat (s**^**-1**^**)**	*K*cat*/Km * **(**μM^**-1**^**s**^**-1**^**)**
SHV-1	49	1460	29.79	26	5910	227.3
SHV-1(L138P)	76	3370	4.43	87	1363	15.66
SHV-33	59	2140	36.27	16	1375	85.93
SHV33-L138P	91	2680	29.45	90	1503	16.7

### Molecular docking simulation of SHV lactamases

**Figure 2:**
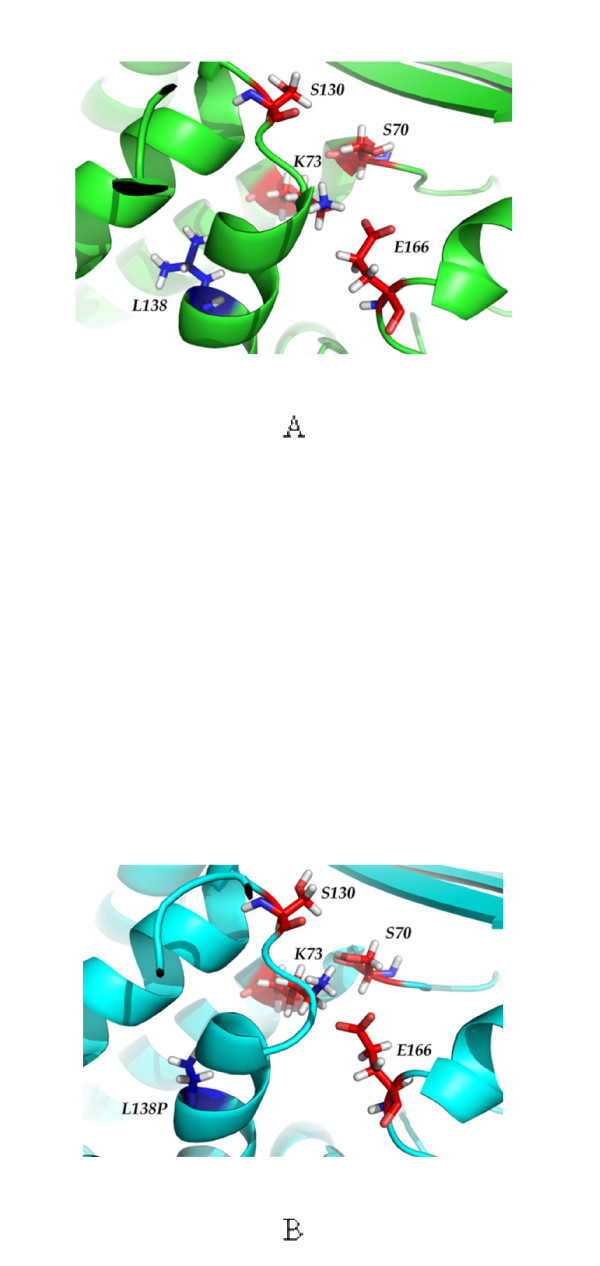
**Structure of the wild-type (A) and L138P β-lactamases (B)**. The red and blue residues indicate the catalytic residues (S70-K73-S130-E166) and mutation site (L138P), respectively.

**Figure 3:**
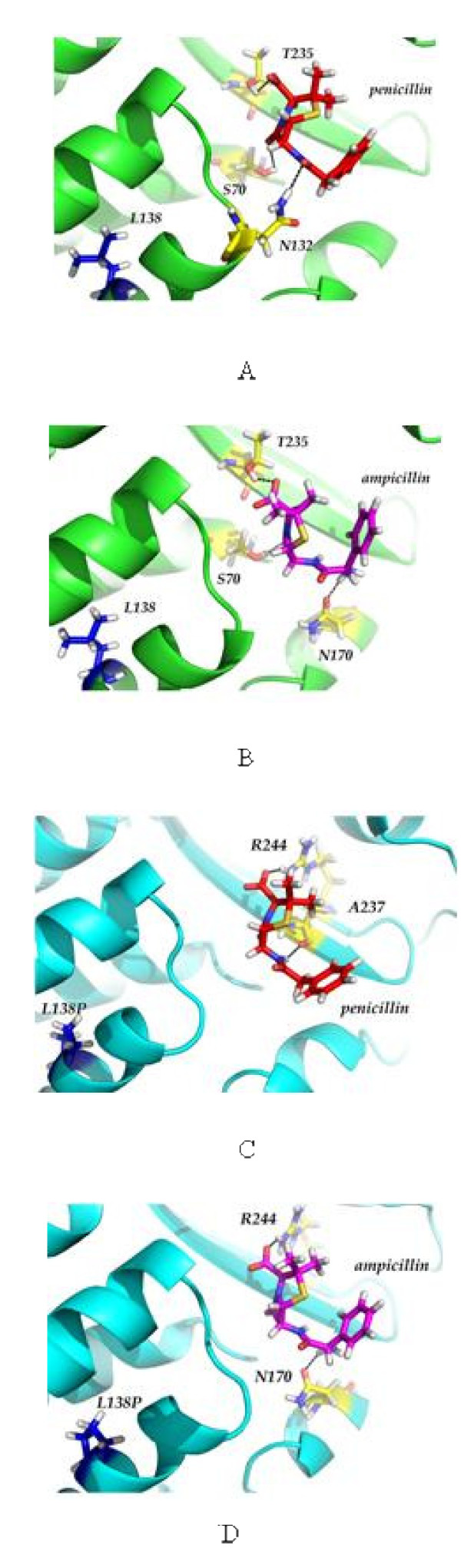
**Modeled docking structures of β-lactamases and penicillin and ampicillin**. (A) Docking structure of the wild-type and penicillin (B) Docking structure of wild-type and ampicillin (C) Docking structure of L138P mutant and penicillin (D) Docking structure of L138P mutant and ampicillin. The dashed lines indicate hydrogen bonds and the red residues indicate catalytic residues.

The structures of the wild-type and L138P mutant were prepared by molecular dynamics. The alpha helix of L138P mutant including 138 position was shorter than that of the wild-type and the orientation of the catalytic residues were slightly changed due to the proline mutation (Figure 2). The productive docking structures with the lowest binding energies predicted by Discovery Studio 2.5 were selected as binding structures of penicillin and ampicillin (Figure 3). The wild-type showed higher binding affinity (lower binding energy) of both penicillin (16.5 kcal/mol) and ampicillin (31.2 kcal/mol) than the L138P mutant, confirming that the L138P mutant had poor binding affinity (higher *K*_*m*_) of penicillin (19.4 kcal/mol) and ampicillin (36.3 kcal/mol) compared to the wild-type. The wild-type and L138P mutant had lower binding energies of penicillin (16.5 and 19.4 kcal/mol respectively) over ampicillin (31.2 and 36.3 kcal/mol respectively), consistent with experimental results that both β-lactamases preferred penicillin to ampicillin.

## Discussion

Extensive research on β-lactam resistance has been carried among the clinical hospital isolates and majority of β-lactamases reported to date have been derived from clinical isolates of humans. However, recent research has shown the increasing occurrence of β-lactam resistance in microbes of animal origin, especially in animal derived *E. coli *and *Salmonella*, which are related to community acquired infections and food safety [1,5,21]. In continuation of our study effort to address some of these issues of antimicrobial resistance in Enterobacteriaceae isolated from farm animals, our study focused on the identification and understanding the dynamics of unique *bla*_SHV-L138P _mutation observed in *E. coli *isolated from swine.

Phenotypic antimicrobial tests showed that the *E. coli *isolate was resistant to the common antimicrobial agents used in farms and also exhibited reduced sensitivity to three indicator cephalosporins included in the study. Genetic analysis showed the presence of both TEM-20 and SHV β-lactamases that differed from SHV-1 only by a single amino acid substitution leucine to proline at position 138. This mutation was of special interest as SHV β-lactamses are specially related to *K. pneumoniae *and we wanted to see if this *bla*_SHV _gene with single amino-acid substitution (L138P) detected in *E. coli *added to its substrate hydrolyzing activity [1,2,4,22,23].

All the cloned *bla*_SHV _genes expressed the specific protein bands that were confirmed by SDS-PAGE and Western blot. The size of the expressed SHV β-lactamases was larger than reported in previous research because of the intact 23 amino acid pro-peptide and His tag [20]. The enzyme kinetics of all the expressed β-lactamases showed differences in the affinities for penicillin and ampicillin that were included in this experiment (Table 3). The narrow spectrum β-lactamases SHV-1 and SHV-33 exhibited higher affinity to penicillin and ampicillin respectively, whereas SHV-1 and SHV-33 with only in one amino acid (L138P) mutation exhibited reduced activity for both the substrate used in study. This indicated that leucine at position 138 was important for SHV β-lactamase and played an important role in hydrolyzing penicillin and ampicilin.

Previous experiments on SHV β-lactamases have reported three natural mutations at position 69, 130 and 187 to be involved in conferring resistance to the inhibitors [11-13]. Proline has stronger stererochemical constraints than any other residues, with only one instead of two variable backbone angles and it lacks the normal amine backbone for hydrogen bonding. This could have the disruptive function to regular secondary structure and decreased the length of α-helix and changed the orientation of residues of binding sites. Based on the modeled docking structures of the wild-type and L138P mutant, the wild-type had three hydrogen bonds with penicillin and ampicillin but the L138P mutant had two hydrogen bonds, indicating that these structural changes by L138P mutation may decrease the substrate binding and finally resulted in reduced activity of L138P mutant. This result was supported by higher *K*_*m *_value for penicillin and ampicillin of L138P mutation when inserted in SHV-1 and SHV-33.

## Conclusions

Based on our results we concluded that this mutation caused a drop in hydrolyzing penicillin and ampicillin. Under the selection pressure imposed by the use of these antimicrobials, naturally occurring L138P mutation in the conserved region of *bla*_SHV _gene was expected to increase substrate hydrolyzing property or widen the substrate spectrum of SHV- β-lactamases. However, adverse effect of this mutation observed on its substrate hydrolyzing properties may be a way these microbes trigger emergence or acquisition of more effective alternative mechanisms. Our speculation is in line with recent reports on CTX-M and AmpC β-lactamases that have more frequently been reported than the classical TEM and SHV β-lactamase from farm and food materials [1,3,4,7,21].

## Authors' contributions

NR, SBC and MKS carried out cloning expression and western blot, SP contributed in enzyme kinetics, JCJ did Simulation docking experiment. YJY and HSY provided guidance and helped coordination. All authors have read and approved the final manuscript.
